# The host-seeking inhibitory peptide, Aea-HP-1, is made in the male accessory gland and transferred to the female during copulation

**DOI:** 10.1016/j.peptides.2011.10.027

**Published:** 2012-03

**Authors:** Chiara Naccarati, Neil Audsley, Jeffrey N. Keen, Jung-Ha Kim, Gareth J. Howell, Young-Joon Kim, R. Elwyn Isaac

**Affiliations:** a Faculty of Biological Sciences, University of Leeds, Leeds LS2 9JT, UK; b The Food and Environmental Research Agency, Sand Hutton, York YO41 1LZ, UK; c School of Life Sciences, Gwangju Institute of Science and Technology, Gwangju 500-712, Republic of Korea

**Keywords:** Aea-HP, *Aedes aegypti* head peptide, SP, sex peptide, SPR, sex peptide receptor, MIP, myoinhibitory peptide, SV, seminal vesicles, MALDI/TOF-MS, matrix-assisted laser desorption ionization time-of-flight mass spectrometry, *Aedes*, Head peptide, Host-seeking, Accessory glands, Seminal fluid, Reproduction

## Abstract

Male accessory glands (MAGs) of insects are responsible for the production of many of the seminal fluid proteins and peptides that elicit physiological and behavioral responses in the post-mated female. In the yellow fever mosquito, *Aedes aegypti*, seminal fluid components are responsible for stimulating egg production, changing female behavior away from host-seeking toward egg-laying and mating refractoriness, but hitherto no behavior-modifying molecule from the MAGs has been structurally characterized. We now show using mass spectrometry and HPLC/ELISA that the MAG is a major site of synthesis of the biologically active decapeptide, Aea-HP-1 (pERPhPSLKTRFamide) that was first characterized by Matsumoto and colleagues in 1989 from mosquito head extracts and shown to have host-seeking inhibitory properties. The peptide is localized to the anterior portion of the MAG, occurs at high concentrations in the gland and is transferred to the female reproductive tract on copulation. Aea-HP-1 has a pyroglutamic acid at the N-terminus, an amidated carboxyl at the C-terminus and an unusual 4-hydroxyproline in position 4 of the peptide. The structure of the peptide with its blocked N- and C-termini confers resistance to metabolic inactivation by MAG peptidases; however the peptide persists for less than 2 h in the female reproductive tract after copulation. Aea-HP-1 is not a ligand for the mosquito sex peptide/myoinhibitory peptide receptor. *A. aegypti* often mate close to the host and therefore it is possible that male-derived Aea-HP-1 induces short-term changes to female host-seeking behavior to reduce potentially lethal encounters with hosts soon after insemination.

## Highlights

► A biologically active peptide (Aea-HP-1) has been chemically identified from *Aedes aegypti* male accessory glands. ► Aea-HP-1 is transferred to the female on copulating. ► Aea-HP-1 is protected from peptide-degrading enzymes of the accessory gland. ► Aea-HP-1 is not a mosquito ‘sex peptide’, but probably alters host-seeking behavior of the post-mated female.

## 1 Introduction

The mosquito, *Aedes aegypti*, is the main insect vector of yellow fever, chikungunya fever and dengue fever viruses in tropical and sub-tropical regions of the world [25]. The close association of *A. aegypti* with urban populations and its changing geographic distribution are contributing to the spread and increased incidence of dengue fever and the life-threatening dengue hemorrhagic fever [40]. Accordingly, there is interest in understanding the factors and mechanisms that determine reproductive success and influence behavior of the biting females, to aid the development of new vector control strategies.

It has been known for a long time that components of seminal fluid made by the male accessory glands (MAGs) and donated to the female during copulation are important for the reproductive success of *A. aegypti*, not only by facilitating the safe transfer of sperm, but also by directly influencing reproductive physiology and diverse behaviors of the post-mated female, including a life-time refractoriness to mating [5–7,20,29]. Mature females couple repeatedly with males, but are in fact monogamous because they become refractory to a second insemination [8]. This refractoriness can be induced by either transplanting intact MAGs from mature males into the thorax of virgin females or by injecting females with a MAG homogenate [14,35]. Other behavioral responses attributed to MAG components in blood-fed female *A. aegypti* include activation of egg development [22], stimulation of oviposition [28] and pre-oviposition behavior [43] and reduction in host-seeking and biting behavior [18]. Surprisingly, the molecules responsible for eliciting these behavioral responses have not been chemically characterized, hindering our understanding the molecular basis of how MAGs modulate the behavior of female mosquitoes. Historically, the attempts at purification of active MAG constituents of mosquitoes have been limited to primitive fractionation techniques and have resulted in confusion about the number and nature of the molecules responsible (for review see [5]). Only recently have advanced analytical techniques been applied to the chemical analysis of *A. aegypti* MAG secretions, but this work has only focused on proteins and not peptides that might be involved in changing the behavior of the female [36,37].

We now report that the MAGs of *A. aegypti* are a source of the head peptide Aea-HP-1 and that the peptide is transferred during copulation to the female reproductive tract. Aea-HP-1 was first isolated from heads and, subsequently, bodies of adult *A. aegypti* and is known to inhibit host-seeking behavior in adult females [4,30,39]. A recent peptidomics study notably failed to identify the source of Aea-HP-1 in endocrine and neuroendocrine cells of adult insects suggesting that the MAG is possibly the principal source of Aea-HP-1 in adults [34].

## 2 Materials and methods

### 2.1 Rearing of insects

*A. aegypti* mosquitoes, originating from the Liverpool School of Tropical Medicine, were reared at a temperature of 26–27 °C and 80–85% relative humidity. Newly emerged adult males and females were maintained together in netted population cages (30 cm^3^) and provided with sterile glucose solution (0.5% w/v) as continual food source. Females at four days old were additionally provided with a meal of murine blood. Eggs were collected from blood-fed females on damp filter paper and kept at 26–27 °C and 82.5% relative humidity. Established procedures were used for culturing larvae [32]. Virgin males and females were collected after placing pupae in individual tubes and were grouped in separate cages with access to glucose until required for either dissection or for mating. *Drosophila melanogaster* were maintained on oatmeal/molasses/agar medium at 25 °C.

### 2.2 Tissue extraction with acidified methanol

Tissues were dissected from adult mosquitoes in phosphate buffered saline (PBS, MP Biomedicals, Cambridge, UK) and collected into acidified methanol (86%, v/v, aqueous methanol and 5% v/v glacial acetic acid). MAGs and male seminal vesicles (SVs) (5 pairs per 100 μl) were typically prepared for analysis by infusing whole tissues in acidified methanol for 30 min, then centrifuging for 10 min at 13,000 rpm in a bench-top microcentrifuge, retaining the supernatant. Homogenization was avoided to provide a cleaner sample for analysis. Reproductive tracts from individual females (virgin or mated females as required) were collected in 25 μl of the acidified methanol and stored at −20 °C until required. The samples were centrifuged as above to provide a clear supernatant for chemical analysis.

### 2.3 Matrix-assisted laser desorption ionization time-of-flight mass spectrometry (MALDI/TOF-MS)

Mosquito tissues were analyzed for Aea-HP-1 by subjecting either acidified methanol extracts or intact tissues to MALDI/TOF-MS analysis. For the methanolic extracts, an aliquot (1 μl) of MassPREP™ MALDI CHCA matrix (Waters Ltd., Manchester, UK) solution (2 mg/ml α-cyano-4-hydroxycinnamic acid in 25% v/v acetonitrile/25% v/v methanol/0.1% v/v trifluoroacetic acid (TFA)) was mixed with 1 μl of peptide sample and applied to a MALDI sample plate. After allowing samples to dry naturally in the air, the dried MALDI plate was transferred to a M@LDI L/R MALDI/TOF mass spectrometer (Waters Ltd.). The instrument used a N_2_ laser at 337 nm; source voltage was set at 15,000 V, pulse voltage was set at 2450 V, reflectron voltage was set at 2000 V, microchannel plate detector voltage was set at 1950 V. Laser energy was set to medium with fine adjustment to optimize signal for each sample. A minimum of 100 laser shots were accumulated and combined to produce a raw spectrum of positive ion monoisotopic peptide masses ([M+H]^+^) within the mass range *m*/*z* 800–4000. Spectra were processed (background subtraction, smoothing and peak centroiding) using MassLynx 4.0 software (Waters Ltd.) and calibrated externally using a datafile obtained for a tryptic digest of yeast alcohol dehydrogenase.

For whole tissue analysis, intact tissues were applied directly to a MALDI sample plate where they were rinsed with water to minimize salt contamination before the application of matrix solution (10 mg/ml α-cyano-4-hydroxycinnamic acid in 50% v/v acetonitrile/0.05% aqueous TFA). Dried samples were then analyzed using a Voyager DE STR MALDI/TOF mass spectrometer (Applied Biosystems, Warrington) as described previously [2]. Spectra represent the resolved monoisotopic [M+H]^+^ masses in positive reflector mode within the mass range *m*/*z* 500–2500. The MALDI laser was directed to areas close to, but not within, the tissue samples to avoid interference with energy transfer during ionization.

Peptide sequence information was obtained by MALDI Post-Source Decay (PSD) analysis of an acidified methanol extract of MAGs and SVs, performed using the Voyager instrument and angiotensin I as the standard for calibration. A PSD spectrum was produced from 7 to 8 spectral segments and stitched together using the Voyager software. Sequences were interpreted manually.

MALDI/TOF-MS of HPLC fractions was performed by drying each fraction and re-dissolving in 10 μl of 70% (v/v) acetonitrile. A 0.5 μl aliquot of the fraction was then added to 0.5 μl matrix and mixed before transfer to a MALDI sample plate. After drying at room temperature, mass spectra were acquired on a Voyager DE STR MALDI/TOF instrument [2].

### 2.4 HPLC (high-performance liquid chromatography) separation of peptides

Samples were diluted 10-fold in 0.1% (v/v) TFA for fractionation by reversed phase high-performance liquid chromatography (RP-HPLC) performed using a System Gold liquid chromatography system (Beckman Coulter UK Ltd., High Wycombe, UK), utilizing a dual pump programmable solvent module 126 and a UV detector module 166 [2]. Samples were loaded via a Rheodyne loop injector onto a Jupiter C_18_ 5 μm 300 Å column (250 mm × 2.1 mm internal diameter) fitted with a 30 mm × 2.1 mm guard column (Phenomenex, Macclesfield, UK). The column was eluted with a linear gradient of 10–60% acetonitrile/0.1% TFA, over 50 min at a flow rate of 0.2 ml/min, and elution monitored at 215 nm. Fractions (0.2 ml) were collected and dried by centrifugal evaporation for immunoassay or mass analysis.

### 2.5 Immunoassay

Peptides were quantified using an indirect enzyme-linked immunosorbent assay (ELISA) for peptides with a C-terminal RFamide, as described previously [1]. Briefly, either HPLC fractions or synthetic Aea-HP-1 (pERPhPSLKTRFamide; pE, pyro-glutamic acid, hP, 4-hydroxyproline; amide, amidated C-terminus) custom synthesized by Biomatik, Cambridge, Canada) were dried onto multiwell plates (Sigma–Aldrich Co., Dorset, UK) at 37 °C, then incubated overnight at 4 °C with 100 μl of 0.1 M bicarbonate (coating) buffer (pH 9.6). Plates were washed three times with 150 μl of 10 mM phosphate–buffered saline 0.1% (w/v) Tween-20 (PBS-T), blocking solution (150 μl; 2% w/v non-fat milk in PBS-T) was added, and the plates incubated for 90 min at 37 °C. After a further PBS-T wash, 100 μl of primary anti-FMRFamide antiserum (Bachem UK Ltd., St. Helens, UK; diluted 1:3000 in PBS-T) was added to each well and the plates incubated for another 90 min at 37 °C. After washing three times with PBS-T, 100 μl of goat anti-rabbit antiserum conjugated with horseradish peroxidase (diluted 1:3000 in PBS-T) was added as secondary antibody and plates were incubated for 40 min at 37 °C. After three PBS-T washes, 100 μl of substrate solution (25 mg O-phenylenediamine, 25 μl H_2_O_2_ in 25 ml 0.1 M citrate buffer, pH 5.0) was added to each well and incubated for 40 min at 37 °C. The reaction was stopped by addition of 50 μl 1.0 N H_2_SO_4_ to each well and optical density was read at 492 nm on a Labsystems Multiskan MCC/340 (ThermoQuest SEG Ltd., Basingstoke, UK).

### 2.6 Immunohistochemistry

MAGs and SVs of male mosquitoes were dissected into ice-cold PBS and fixed in 4% (w/v) paraformaldehyde in PBS at 4 °C overnight. Fixed tissues were washed four times in PBS before incubation for 2 days at 4 °C with anti-FMRFamide primary antibody diluted 1:500 in 0.3% v/v Triton X-100 in PBS (TX-PBS) containing 2% v/v goat serum). A control was performed by incubating fixed tissue with 2% (v/v) goat serum in TX-PBS without the primary antibody. Excess reagent was washed away with TX-PBS (4 × 15 min) before incubating samples for 2 days at 4 °C with secondary antibody (Alexa Fluor 546 goat anti-rabbit IgG, Invitrogen, Paisley, UK). Secondary antibody was diluted 1:500 in TX-PBS containing 2% v/v goat serum. A further control was performed by pre-incubating 250 μl of secondary antibody (diluted 1:500 in TX-PBS containing 2% v/v goat serum) with 25 μl of 1 mM Aea-HP-1 prior to incubation with tissue. Excess reagent was washed away with TX-PBS (4 × 15 min) before mounting tissue on slides for confocal microscopy. Mounting was performed in 4,6′-diamidino-2-phenylindole (DAPI) diluted 1:1000 in Vectashield^®^ Mounting Medium (Vector Laboratories Ltd., Peterborough, UK). Slides were stored in the dark at 4 °C overnight before microscopic examination. Images were captured using an inverted LSM510 META laser scanning confocal (Carl Zeiss) microscope. Pinholes were set to 1 Airy Unit which gave a 1 μm optical section with a 40× oil immersion objective. Alexa Fluor 546 was excited with the 543 nm HeNe laser and emission was collected through a long pass LP560 emission filter. DAPI was excited with a 405 nm laser diode and emission was collected through a LP420 emission filter. For determining the volume of the MAG, the gland surface was non-specifically coated with Alexa Fluor 546 goat anti-rabbit IgG and serial optical z-sections were collected using confocal microscopy as described above (omitting the collection of the DAPI channel) through the full depth of the gland with z-steps of 0.5 μm. Approximately 60–80 images were required to image the full volume of the MAG. Image stacks were then imported into Imaris software (version 5.7, Bitplane AG, Zurich, Switzerland). Using the Surface Contour tool within Imaris we delineated the outside surface of each slice manually and rendered the images to give us a three dimensional model from which we could determine the gland volume. We analyzed 4 glands in this manner.

### 2.7 Peptidase activity

MAGs were homogenized in 0.2 M HEPES buffer, pH7, 0.2% Triton X-100 (15 pairs of glands in 150 μl). Aliquots (10 μl) were incubated with 1.25 nmoles of peptide at 25 °C. Reactions were stopped by addition of 260 μl of 0.1% TFA and the amount of parent peptide remaining was quantified by reversed phase HPLC [17].

### 2.8 Injection of Aea-HP-1

Injections of either Aea-HP-1 or SP into the abdomen of virgin *D. melanogaster* females were performed as described previously [42]. After injection, females were transferred to individual food vials and were tested after 5 h for receptivity with a naive Canton S male.

### 2.9 Functional expression of sex peptide receptors

Ligand-mediated receptor activation was measured using an established expression system employing CHO-K1 cells expressing the Ca^2+^ reporter aequorin [42]. The construction of the expression constructs for *D. melanogaster* sex peptide receptor (SPR) and *A. aegypti* SPR and the measurement of the luminescent signals have been reported previously [19].

## 3 Results

### 3.1 Identification of Aea-HP-1 as a major peptide component of the *A. aegypti* MAG

**Fig. 1:**
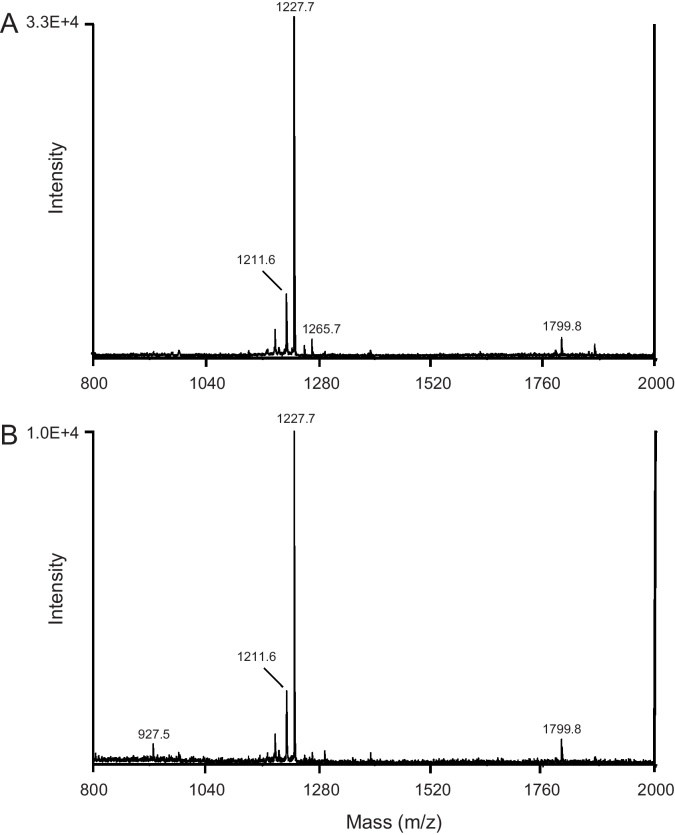
MALDI/TOF mass spectra of a peptide extract of MAGs/SVs (A) and whole MAGs (B). (A) Five pairs of tissues were extracted into acidified methanol (0.1 ml) of which 1 μl of the sample was mixed with 1 μl MALDI matrix solution and dried on a MALDI plate for analysis. The spectrum shows the presence of prominent peaks at *m*/*z* 1211.8 and 1227.8. (B) A pair of MAGs from a single male mosquito were placed directly on a MALDI plate, rinsed with water to eliminate salts, mixed with matrix solution, dried and analyzed as described in Section 2.

**Fig. 2:**
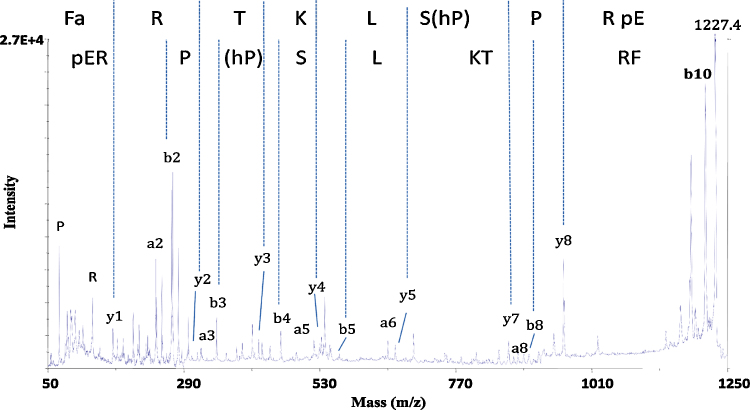
MALDI-PSD fragmentation spectra of the peak at *m*/*z* 1227.8 and synthetic Aea-HP-1. The molecular ion *m*/*z* 1227.8 was subjected to PSD analysis. The final spectrum was constructed from 7 to 8 spectral segments, stitched together using the Voyager software and interpreted to enable sequence determination; synthetic Aea-HP-1 was also subjected to MALDI-PSD, giving an almost identical fragmentation spectrum (not shown).

Peptides were extracted from MAGs plus SVs for analysis by MALDI/TOF-MS. The spectrum (*m*/*z* 800–4000) revealed two prominent monoisotopic peaks, one at *m*/*z*, 1227.8 and a less intense peak at *m*/*z*, 1211.8 (Fig. 1a). The mass difference of 16 Da between these peaks suggested they might be related, with a difference in oxidation state between them. The molecular ion (*m*/*z*, 1227.8) was subjected to post-source decay analysis and the fragmentation spectra generated revealed the amino acid sequence of the parent peptide as pERPhPSLKTRFamide (pE, pyro-glutamic acid, hP, 4-hydroxyproline; amide, amidated C-terminus; Fig. 2). The sequence was identical to a neuropeptide previously isolated from *A. aegypti* heads collected from a mixed sex population and known as the head peptide or Aea-HP-1 [30]. Aea-HP-1 is modified post-translationally in three places, including hydroxylation of one proline residue. The fully modified mature peptide has a theoretical molecular mass ion *m*/*z* of 1227.7, which agrees closely with the *m*/*z* peaks observed in our spectra. The second peak at *m*/*z*, approx. 1211.7 most likely represents a version of the peptide known as Aea-HP-3 [39], in which the proline at position four was not hydroxylated. An almost identical fragment ion spectrum was generated when synthetic Aea-HP-1 was analyzed in the same manner (not shown). An extract from a total of 70 pairs of MAGs and SVs was fractionated using RP-HPLC and MALDI/TOF-MS analysis of collected fractions established that the retention times of the natural and synthetic Aea-HP-1 were identical.

MAGs and SVs were also analyzed separately by directly placing tissues from individual insects onto the MALDI plate. The prominent ions previously observed in the acidified methanol extract were also the major peaks (*m*/*z*, 12211.6 and 1227.6, Fig. 1b) in the spectra obtained directly from pairs of MAGs and were consistently seen in tissues from seven individual mosquitoes. In contrast, the same two mass ions (spectra not shown) were only detected in three out of seven pairs of SVs, suggesting that the MAGs were probably the main source of Aea-HP-1.

### 3.2 Aea-HP-1 is localized to the anterior region of the MAG

**Fig. 3:**
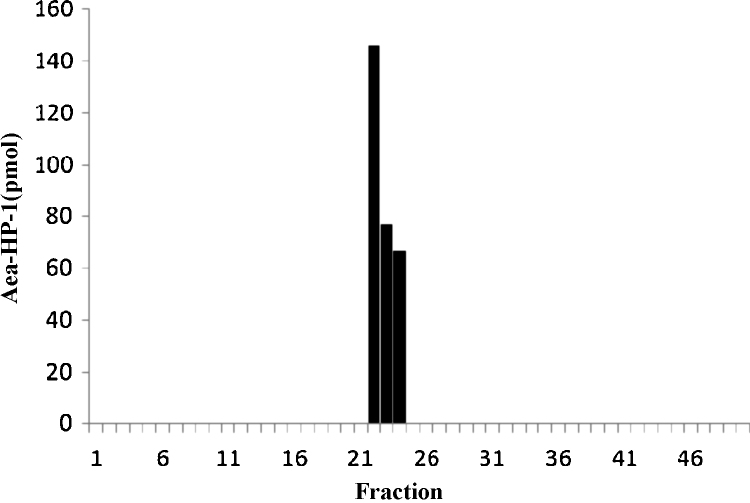
Co-chromatography of FMRFamide immunoreactivity with synthetic Aea-HP-1. An acid–methanol extract of MAGs was fractionated using RP-HPLC. The elution of Aea-HP-1 was monitored using both MALDI/TOF-MS and ELISA for FMRF-amide-like peptides. Aea-HP-1 was detected by MALDI/TOF-MS (molecular ion, *m*/*z* 1227.6) in fractions 22–24 which corresponded to the retention time of synthetic Aea-HP-1. The same fractions were the only ones that gave an above background response in the ELISA. The calibration plot for Aea-HP-1 was linear between 10 and 1000 fmol.

Aea-HP-1 cross-reacts with antibodies raised to the invertebrate peptide, FMRFamide, presumably because of the common RFamide epitope [4,30,39]. We therefore used a commercially available FMRFamide antibody in an ELISA to monitor HPLC fractions for Aea-HP-1 and any other FMRFamide-like peptides that might be present in the MAGs/SVs extract (Fig. 3). A single peak of ELISA-positive material was eluted from the HPLC column in three consecutive fractions that were also positive for mass ion *m*/*z* of 1227.7. All other UV-absorbing fractions gave negative ELISA results, suggesting that Aea-HP-1 was the major peptide component of the extract displaying cross-reactivity to the FMRFamide antibody.

**Fig. 4:**
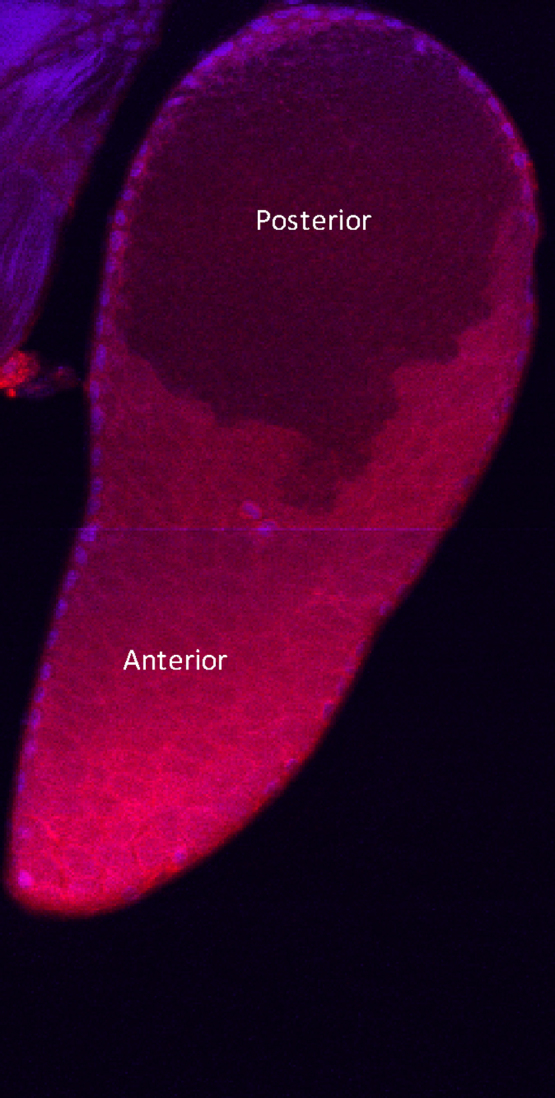
Confocal microscopy of MAGs and SVs from *A. aegypti*. Whole MAGs were fixed and then treated with primary FMRFamide antibody and detected using a fluorescent secondary antibody, as described in Section 2; counter-staining was with DAPI. The gland is structurally divided into a posterior part and anterior part, where the peptide appears concentrated. No staining was observed when primary antibody was pre-blocked with Aea-HP-1 or when primary antibody was omitted.

Confocal microscopy of whole mounted MAGs/SVs stained using the same antibody revealed differential distribution of the immunoreactivity with staining largely restricted to the MAGs and much of this being concentrated in the lumen of the anterior region of the gland (Fig. 4). During the course of this investigation it was noticed that dissected MAGs with SVs attached often released gland contents from the SVs by spontaneous contractions of the MAG muscle layer. It was therefore possible to collect MAG secretions into PBS using a pipette and place this material into acidified methanol. Both Aea-HP-1 and Aea-HP-3 were detected in these secretions by MALDI/TOF-MS (*m*/*z*, 1227.8 and 1211.8, respectively), confirming the presence of these peptides as components of the MAG secretions and therefore seminal fluid.

### 3.3 Copulation results in the transfer of Aea-HP-1 from the male to the female reproductive tract

MALDI/TOF-MS was used to analyze methanol extracts for the presence of Aea-HP-1 in the reproductive tract (uterus, spermathecae, bursa copulatrix, oviduct, but not the ovary) of individual virgin and post-mated females obtained by introducing a single female into a cage of 50 males until mating was observed (<5 min). In order to minimize clearance of any transferred Aea-HP-1 from the female reproductive tissues, post-mated females were chilled to 4 °C immediately after copulation. Mass spectrometry failed to detect any evidence for Aea-HP-1 in virgin tissues from ten individual mosquitoes. In contrast, the molecular ions for Aea-HP-1 (*m*/*z*, 1227.9) and Aea-HP-3 (*m*/*z*, 1211.9) were detected in methanol extracts of tissues from nine out of thirteen post-copulated females. Extracts were also analyzed quantitatively by ELISA using synthetic Aea-HP-1 as a standard and the results were compared to the amount of material found in extracts of MAGs dissected from virgin males. Peptide was detectable in reproductive tissues for ten out of thirteen post-copulated females with a mean value of 102 ± 14 fmol of peptide/insect (mean ± s.e.m., *n* = 10), whereas the level of peptide in the reproductive tract of all 10 virgin females was below the detection level of the ELISA (<10 fmol). Pairs of MAGs from unmated males contained much greater amounts of peptide (1311 ± 232 fmol, mean ± s.e.m., *n* = 10). In a separate study, post-mated females were kept at 26 °C for different time periods (0.5 h and 2 h) before the reproductive tissues were removed for extraction and analysis by MALDI/TOF-MS. Aea-HP-1 was detected in tissues from all 0.5 h post-mated females (*n* = 15, but only 1 out of 10 samples for the 2 h post-mated females.

We used confocal microscopy to determine the volume of a single MAG as 1.67 ± 0.08 nl (mean ± s.e.m., *n* = 4), which allowed us to estimate the Aea-HP-1 concentration in the MAGs to be around 400 μM.

### 3.4 Stability of Aea-HP-1 to exposure to peptide-degrading MAG peptidases

**Fig. 5:**
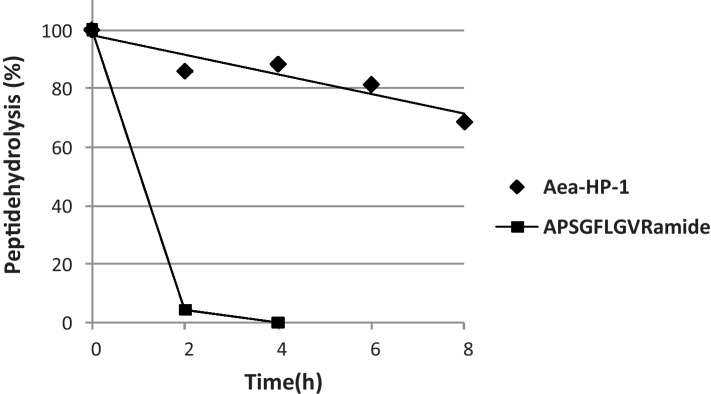
Relative stability of Aea-HP-1 and APSGFLGVRamide to degradation by MAG peptidases. The stability of each peptide on incubation with a homogenate of MAGs was monitored by quantification of Aea-HP-1 and APSGFLGVRamide using HPLC with UV-detection (214 nm).

Reproductive tissues of *A. aegypti* are known to be rich in peptidases that might be involved in the metabolism of MAG peptides [37]. We confirmed the presence of peptide-degrading peptidases using the insect peptide, APSGFLGVRamide, as a substrate. Under conditions that resulted in over 96% hydrolysis of APSGFLGVRamide, only 8% of Aea-HP-1 was degraded, demonstrating the relative stability of Aea-HP-1 to MAG enzymes (Fig. 5).

### 3.5 Is Aea-HP-1 a functional homologue of the sex peptide of *D. melanogaster*?

**Fig. 6:**
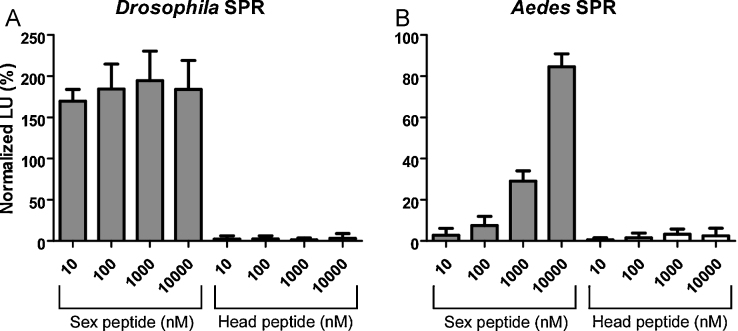
Aea-HP-1 is not an active agonist for SPRs from *D. melanogaster* (A) and *A. aegypti* (B). Luminescent responses of CHO cells expressing SPR, aequorin and a chimaeric G-protein, Gα_qi_, and treated with varying concentrations of Aea-HP-1 and SP. Each data point is mean ± s.d. (*n* = 4).

**Fig. 7:**
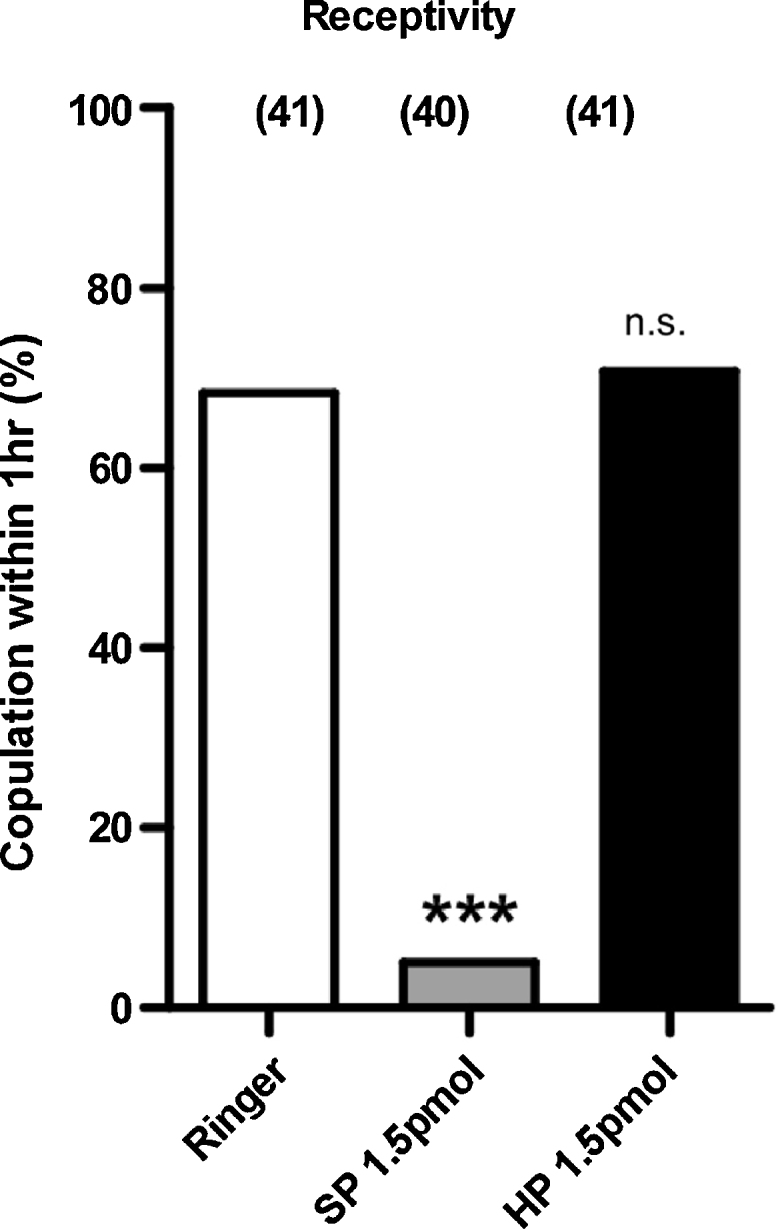
Aea-HP-1 injection does not induce the post-mating response in *D. melanogaster*. Receptivity of virgin females assayed 5 h after injection with either synthetic peptides (Aea-HP-1 or SP) or Ringer's solution alone. Wild type W1118 females were injected and assayed with naïve Canton-S males. Numbers in parenthesis indicate numbers of animal assayed. n.s., *p* > 0.05; ***p < 0.0001, one-way ANOVA (Tukey post-test) compared with Ringer treatment.

The most studied peptide of insect MAGs is the sex peptide (SP) of *D. melanogaster.* This 36 amino acid peptide has not been found outside of a sub-group of closely related *Drosophilidae*. It has multiple signaling roles in the post-mated female, the best known of which is a decrease in sexual receptivity to courting males. Recently, it has been shown that SP and insect myoinhibitory peptides (MIPs) are ligands for the same G-protein coupled receptor despite lack of structural similarity; MIPs, like Aea-HP-1, are relatively short peptides (generally 9–12 amino acids) with an amidated C-terminus. This promiscuity of the SP/MIP receptor led us to test whether Aea-HP-1 might be an additional agonist for this receptor. We therefore carried out experiments to see if Aea-HP-1 could elicit a post-mating response in virgin female *D. melanogaster* (Fig. 6) [42]. We also tested directly whether Aea-HP-1 was an agonist of the SP/MIP receptor of either *D. melanogaster* or *A. aegypti* using an established cell-based assay for receptor activation (Fig. 7) [19]. Aea-HP-1 did not elicit rejection of male advances when injected into the hemocoel of virgin *D. melanogaster* females and did not activate the SP/MIP receptors up to 10 μM.

## 4 Discussion

We have for the first time chemically characterized a peptide (Aea-HP-1) with biological activity from the MAG of a mosquito and shown that this molecule is transferred to the female on copulation. Aea-HP-1 is a ten amino acid peptide that was first isolated from >600,000 heads of mixed-sex mosquitoes in 1989 together with the tripeptide Aea-HP-2 (TRFamide) using a radioimmunoassay for the molluscan peptide FMRFamide to guide purification [30]. Aea-HP-3 and a pentapeptide C-terminal fragment (Aea-HP-4) were subsequently found in extracts of the abdomen of adult *A. aegypti* in addition to Aea-HP-1 [39]. Both N-terminally truncated forms of the head peptides (Aea-HP-2 and Aea-HP-4) are probably generated by proteolysis of the decapeptides. Our MALDI/TOF-MS analysis showed that both Aea-HP-1 and Aea-HP-3 are present in the MAGs and HPLC analysis combined with MALDI/MS and ELISA indicated that Aea-HP-1 is the dominant form. The hydroxylation of Pro in biologically active peptides is unusual and, as far as we are aware, occurs in only three other insect peptides, one of which, interestingly, is the SP of *D. melanogaster* [10,11] and the others being [Hyp3]Met-callatostatin and [Hyp2]Met-callatostatin of the blowfly [11,12]. Aea-HP-1 and Aea-HP-3, like many insect regulatory peptides, have an amidated C-terminus and a pyroglutamate at the N-terminus, both modifications render peptides more resistant to degradation by exopeptidases [16]. Resistance to hydrolysis by peptidases will be important for maintaining biological activity during transfer to the female since the MAGs and seminal fluid of *A. aegypti* are known to contain several exopeptidases [36]. Indeed, we have shown in the present study that MAGs contain peptide-degrading peptidase activity and that Aea-HP-1 is relatively stable in the presence of these hydrolytic enzymes.

Aea-HP-1 has been tested for myogenic and behavior modifying activity in *A. aegypti*. The peptide did not stimulate contractions of isolated oviduct and hindgut of female mosquitoes [31], but did alter behavior when injected into non-öogenic females by inhibiting host-seeking behavior [4]. This reduction in host-seeking lasted for up to 5 h and the effect was possibly time limited by the rapid clearance of the peptide from the mosquito hemolymph – only around 17% of the peptide remained in the circulation after 30 min [4]. Aea-HP-3 did not elicit host-seeking inhibitory behavior when injected into females indicating that the presence of a hydroxyl group on Pro^4^ is important for this activity [4].

MAGs of *A. aegypti* are composed of a thin muscle sheaf surrounding a single layer of secretory cells that form distinct anterior and posterior regions with different modes of secretion [9]. Immunohistochemistry using antibodies that cross-react with Aea-HP-1 identified the anterior region of the MAG as the likely source of the peptide. These cells make up around two-thirds of the MAG and release their contents into the lumen by an apocrine mechanism involving the pinching off of apical parts of the cell [9]. Aea-HP-1 is generated by limited proteolysis of the preprohormone that comprises a secretory signal peptide and three copies of the peptide precursor sequence [38]. Further post-translational processing will generate either Aea-HP-1 or Aea-HP-3. We were able to detect Aea-HP-1 in fluid emanating from the MAGs, indicating that the peptide is present in the secretions and is a component of the seminal fluid that is eventually passed to the female during mating. This was confirmed by demonstrating that Aea-HP-1 is present in the female reproductive tissues soon after copulation, but not in tissues of virgins. We had difficulty detecting Aea-HP-1 peptide in the female tissues at 2 h post-copulation, possibly because of transfer from the female reproductive tract into the hemolymph in a similar manner to that proposed for SP in post-mated females of *D. melanogaster*. Fernandez and Klowden compared the hemolymph titers of Aea-HP-1 in gravid unmated and mated female *A. aegypti* using a radioimmunoassay [13]. The results suggested higher levels of Aea-HP-1 in the mated females, although the difference was not statistically significant and the identity of the immunoreactivity was not confirmed by chromatography.

Recently, a mass spectrometric study conducted by an expert group in the field of insect neuropeptidomics compiled a comprehensive list of mature neuroendocrine peptides present in the central nervous system, neurohemal organs and various endocrine cells (including those of the mid-gut) of *A. aegypti*, but failed to detect Aea-HP-1 (or Aea-HP-3) in any of these tissues [34]. It is therefore not possible to pinpoint the source of Aea-HP-1 that was originally isolated from a large quantity of heads. These results taken together with the ease in which we detect Aea-HP-1 in a single MAG suggest that the male reproductive tissue is a major site of synthesis of this peptide, at least in adult males. None of the head peptides have been found in the peptidomes of other insects and genes coding for the head peptides have been notably absent from insect genome databases, even those of other mosquitoes, suggesting a specific role for the peptide in reproduction of *A. aegypti*. Aea-HP-1 might fall into the category of male reproductive proteins which are subject to much faster evolutionary change compared to proteins of non-reproductive tissues, possibly driven by sexual conflict [3].

Transplantation of MAGs, or injection of gland extracts, into female *A. aegypti* can elicit a variety of post-mating responses including refractoriness to re-mating and inhibition of host-seeking and biting behavior [13]. We have not been able to show that Aea-HP-1 can induce mating rejection by injecting Aea-HP-1 (0.1–1 μg) into the hemocoel of virgin *A. aegypti* females (not presented). This failure might be due to lack of accessibility and the known rapid catabolism of Aea-HP-1 in the hemolymph [4]. *D. melanogaster* SP has wide-ranging effects on physiology and behavior some of which are mediated primarily through activation of the SP/MIP receptor that is expressed in sensory neurons of the uterus [42]. The *D. melanogaster* SP receptor and its *A. aegypti* orthologue are also strongly activated by myoinhibitory peptides (MIPs), a family of peptides that are found in species from different insect orders and are considered to be the ancestral ligands for these receptors [19,33,41]. The receptors are also activated by unidentified ligands present in whole body extract of adult *A. aegypti* [19]. Although Aea-HP-1 has no obvious structural similarity to either SP or MIP, the promiscuity of the SP/MIP receptor led us to consider the possibility that the head peptide might also be an agonist of the MIP receptor. However, Aea-HP-1 did not activate the mosquito SP/MIP receptor in a well-established *in vitro* assay for receptor activity.

Aea-HP-1 appears to have a role in changing the behavior of female *A. aegypti* after a blood-meal. Females refrain from host-seeking in two phases; within 1 h after a blood-meal [23] and a second phase starting 30 h post-blood-meal which continues until oviposition and the start of another gonadotrophic cycle. The first loss of interest in a host is triggered by distension of the abdomen [24] and the later sustained response to the blood-meal appears to involve the release of Aea-HP-1-like material into the hemolymph at around 24 h after the meal from either neurosecretory or midgut endocrine cells [4]. Changes in host-seeking behavior in response to a blood-meal are strongly influenced by the size of the meal and whether the female has mated [21]. Gravid females are more likely to desist from seeking a host if they have been inseminated [18,23,24,26,27]. Lavoipierre showed that biting by gravid virgin *A. aegypti* females with developing öocytes (fifth stage) was rapidly and completely inhibited by mating and that this effect lasted for around 4–5 h, suggesting the existence of a fast acting inhibitory factor [27]. Implantation of MAGs or injection of a MAG homogenate into virgin gravid females results in inhibition of host-seeking and feeding, suggesting that substances made in the MAG and presumably present in seminal fluid are involved in changing female behavior toward the host [13,18]. The ability of the male to influence inseminated gravid females in this way is possibly an adaptation that helps to minimize risks from defensive actions of a host (see [21]). Gravid females who have not yet mated might benefit from maintaining host-seeking behavior because in the natural environment sexually competent males are also attracted to the host, thus increasing the chances of mating success [15]. Our discovery that high concentrations of Aea-HP-1 are found in the MAG and that the peptide is transferred to the female suggests a mechanism by which the male can influence the behavior of the female either by activating sensory neurons in the female reproductive tract or by elevating Aea-HP-1 levels in the hemolymph.
